# Comprehensive analysis of Twitter usage during a major medical conference held virtually versus in-person

**DOI:** 10.1186/s13244-021-01140-0

**Published:** 2022-01-20

**Authors:** Nedim Christoph Beste, Xue Davis, Roman Kloeckner, Erkan Celik, Michael Korenkov, David Maintz, Thomas Dratsch, Daniel Pinto dos Santos

**Affiliations:** 1 Institute of Diagnostic and Interventional Radiology, University Hospital of Cologne, Kerpener Strasse 62, 50937 Cologne, Germany; 2 Department of Psychiatry, Yale University School of Medicine, 1 Church St. Ste 6A, New Haven, CT 06510 USA; 3 Department of Diagnostic and Interventional Radiology, University Medical Center of the Johannes Gutenberg University Mainz, Langenbeckstraße 1, 55131 Mainz, Germany; 4 Faculty of Medicine and University Hospital of Cologne, Institute of Virology, University of Cologne, Kerpener Strasse 62, 50937 Cologne, Germany

**Keywords:** Twitter, Social media, Medical conferences, RSNA, Science communication

## Abstract

**Background:**

Twitter has become one of the most important social media platforms in science communication. During scientific conferences, Twitter can facilitate the communication between audience and speakers present at the venue and can extend the reach of a conference to participants following along from home. To examine whether Twitter activity can serve as a surrogate parameter for attendance at the RSNA conferences in 2019 and in 2020, and to characterize changes in topics discussed due to the virtual character of the 2020 RSNA conference.

**Methods:**

The Twitter API and R Studio were used to analyze the absolute number and frequency of tweets, retweets, and conference-related hashtags during the 2019 and 2020 RSNA conference. Topics of discussion were compared across years by visualizing networks of co-occurring hashtags.

**Results:**

There was a 46% decrease in total tweets and a 39% decrease in individual Twitter users in 2020, mirroring a 43% decrease in registered attendees during the virtual conference. Hashtags related to social initiatives in radiology (e.g., “#radxx” and “#womeninradiology” for promoting women’s empowerment in radiology or “#pinksocks,” “#weareradiology” and “#diversityisgenius” for diversity in general) were less frequently used in 2020 than in 2019.

**Conclusion:**

Twitter and congress attendance were highly related and interpersonal topics underwent less discussion during the virtual meeting. Overall engagement during the virtual conference in 2020 was lower compared to the in-person conference in 2019.

## Keypoints

- Twitter activity and attendance on medical conferences are highly related.
- Twitter activity was lower during a virtual compared to an in-person conference.
- Interpersonal topics were less discussed during a virtual conference.

## Background

Since its founding in 2006, Twitter has become one of the most important social media platforms in healthcare communication [1]. Recently, the percentage of healthcare providers and scientists on Twitter in radiology [2, 3] and other medical specialties [4–6] has sharply increased. Organizing social and political initiatives (e.g., using the hashtag “#radxx” for women’s empowerment in radiology) and staying up to date in an individual field of interest [6–8] are among the many uses that make Twitter an attractive tool for those in the healthcare field.

Twitter has also become very popular at conferences for medical specialties, including radiology [9–14], for myriad reasons. Through Twitter speakers can directly connect with their audience by conducting live polls, attendees can share their impressions by live-tweeting (posting content about an event while attending it), and people not able to attend the conference can follow along from home. Consequently, it is increasingly common for medical conferences to create dedicated hashtags (e.g., #RSNA2019 for the RSNA’s annual meeting in 2019) in order to facilitate aggregating all posts related to that particular conference [9, 13, 15–17].

Up until 2020, Twitter activity during RSNA Annual Meetings has grown continuously. For example, an analysis of meeting-related hashtags at RSNA 2011 and RSNA 2012 showed an increase in Twitter usage of about 30% [18]. However, due to the coronavirus pandemic, most scientific conferences in 2020 were held exclusively online as virtual conferences. This had a direct effect on the number of attendees. For instance, at the RSNA Annual Meeting, the number of registered attendees decreased from 51,800 in 2019 [19] to 29,339 in 2020 [20].

Initially, we were interested in how Twitter might reflect the development of specific interests and topics (e.g., artificial intelligence) at RSNA conferences over the years. However, as RSNA was held virtually in 2020, we took the opportunity to analyze the impact of the virtual setting on engagement and topics under discussion.

## Materials and methods

### Study design and data collection

Tweets and their corresponding metadata were collected once a day during the 105th (Dec 1–Dec 6, 2019) and the 106th (Nov 29–Dec 5, 2020) RSNA Annual Meetings, as well as during the 3 days before and after each conference, by accessing the Twitter application programming interface (Twitter API) with the retweet library for R (version 4.0.5). Specifically, R Studio (Version 1.4.1106), a more user-friendly version of the statistic program R, was used to access the Twitter API and conduct the analysis. The Twitter API provides the opportunity to access Twitter data such as tweets, hashtags and user information (e.g., username, location). Relevant tweets were identified using the given congress hashtags (#rsna19 & #rsna2019 and #rsna20 & #rsna2020) and indexed with their unique Tweet ID. Importantly, all hashtags were set to lowercase before analysis. Simple retweets with no additional original text were excluded. Parameters collected included user information such as username, number of followers, and favorite counts; as well as the date and text of the tweet along with included hashtags.

### Data analysis

Using R Studio, we performed descriptive analyses of meeting-related Twitter activity including number of tweets, retweets and favorites. We also examined metrics of individual Twitter users engaging with the conference, such as their number of followers and friends. “Followers” refers to people who subscribe to, or follow, an individual’s Twitter account who are then shown that person’s tweets in their feed; and “friends” refers to people that a Twitter user follows. We determined the number of friends and followers at the time of each tweet, as well as computed the median number of friends and followers for each user over 1 year. Also, changes in total number of friends and followers during the time course of the conferences were calculated. For qualitative analyses, we examined hashtags, username, and tweet source using R Studio. Hashtags were converted to lower case and then counted and sorted by frequency. To account for the unequal sample size, individual hashtags were set in relation to the total number of conference-related tweets in each respective year. Only hashtags that were used an average of 50 times or more in both years were included in the analysis. In addition, we examined the change in frequency of use for each hashtag and investigated the 75 most frequently co-occurring hashtag pairs across years. Furthermore, we examined which application was used to post each tweet and sorted them by primary application environment (non-mobile, mobile, or both)**.** “Non-mobile” refers to apps used on stationary devices (e.g., Twitter Web Client on a desktop) while “mobile” refers to apps used on smartphones and tablets (e.g., Twitter applications for iPhone, Android or iPad). “Both” refers to apps that could be used on both types of devices. To examine differences in user profiles across years, the 50 most active users of each year were manually sorted into “business,” “personal,” and “education/non-profit” categories. Accounts were considered business users when they represented a company and not personal opinion (e.g., @CanonMedicalUS). In contrast, accounts were considered “personal” if individual users tweeted a personal opinion (e.g., residents or employees of medicine-related companies). All accounts belonging to universities, institutions or non-profit organizations (e.g., @RSNA, @DukeRadiology) were sorted in the category “educational/non-profit.” The proportion of tweets in each category was compared.

### Statistical analysis

Using R Studio, differences in application environment were analyzed using chi-squared tests using Cramer’s V as the effect size. Robust ANOVA was used to examine categories. To compare counts of favorited tweets, retweets, friends, and followers across years, we used two-sample Wilcoxon Rank Sum tests after ruling out normal distribution using Shapiro–Wilk tests. The effect size *r* was used for the Wilcoxon–Rank-test. *P* values < 0.05 were considered significant. Results are given as mean ± standard deviation unless otherwise noted. All statistical analyses were conducted using RStudio Version 1.4.1103.

## Results

### Quantitative analysis

Table 1 gives an overview of our quantitative analyses. More tweets were sent in 2019 (11,880 tweets) than in 2020 (6770 tweets). Moreover, the number of individual Twitter users actively participating in the conference decreased from 2076 (2019) to 1276 (2020). On average, there were more retweets per tweet in 2019 compared to 2020 (*M* = 1.70 ± 3.61 vs. *M* = 1.44 ± 4.17; *Z *= − 9.23; *p* < 0.001; *r* = 0.05). Furthermore, Wilcoxon Rank Test revealed a difference in the mean number of favorites per tweet in 2019 compared to 2020 (2019: *M* = 7.41 ± 16.4; 2020: *M* = 7.79 ± 17.5; *Z* = − 3.12; *p* = 0.002; *r* = 0.02). Additionally, we found that in both years 94% of tweets had less than 30 retweets and favorites, indicating that only a small percentage of tweets had broad reach**.**

**Table 1:** Overview of the Twitter activity at the in-person RNSA conference in 2019 and the virtual RSNA conference in 2020

	2019	2020
Tweets		
Total tweets	11,880	6770
Individual users	2076	1276
Mean retweets per tweet	1.7 ± 3.61	1.4 ± 4.17
Mean favorites/likes per tweet	7.4 ± 16.4	7.8 ± 17.5
Tweet sources		
Mobile users	53%	32%
Non-mobile users	17%	44%
Mobile/non-mobile users	27%	23%
Top 50		
Private users	38%	36%
Business users	21%	20%
Educational/institutional users	40%	44%

Top 50: 50 most active Twitter users per year (i.e., users who posted the most tweets)

### Top 50 Twitter users

We focused on the 50 most active Twitter users (Top 50) engaging in the conference each year in order to examine differences in user profiles across years. Not surprisingly, we observed a higher number of tweets per user among the Top 50 in 2019 than in 2020 (2019: total tweets = 4043; 2020: total tweets = 2939; *Z* = − 3.53; *p* < 0.001; *r* = 0.25**)**. While they represented 2% (2019) and 5% (2020) of total users, respectively, their tweets reflected 34% (2019) and 48% (2020) of total tweets. We also calculated how many followers and friends the Top 50 gained during the meeting in both years. Interestingly, the Top 50 acquired more new followers in 2019 than in 2020 (2019: *M* = 48.8 ± 68.9; 2020: *M* = 32.8 ± 54.5; *Z* = − 2.80; *p* ≤ 0.01; *r* = 0.2). In addition, the number of private, business and educational/non-profit users changed significantly across years (*N* = 7861, *X*^2^ = 9.5, *p* < 0.01; *r* = 0.03): We observed more educational/non-profit users (2019: 40%, 2020: 44%) and less private (2019: 38%, 2020: 36%) and business (2019: 21%, 2020: 20%) users among the Top 50 in 2020.

### Tweet sources

We next examined tweet source, focusing on whether an application used to post a tweet was more likely installed on a mobile or a non-mobile device. We detected that in 2019 more tweets were sent from mobile devices (6313 tweets (53%)) than in 2020 (2147 tweets (32%)), while the percentage of tweets sent from non-mobile devices was lower in 2019 (2019: 17%; 2020: 44%; *N* = 18,650, *X*^2^ = 1553, *p* ≤ 0.001, Cramer’s *V* = 0.29) (Table 1).

### Hashtags

**Fig. 1:**
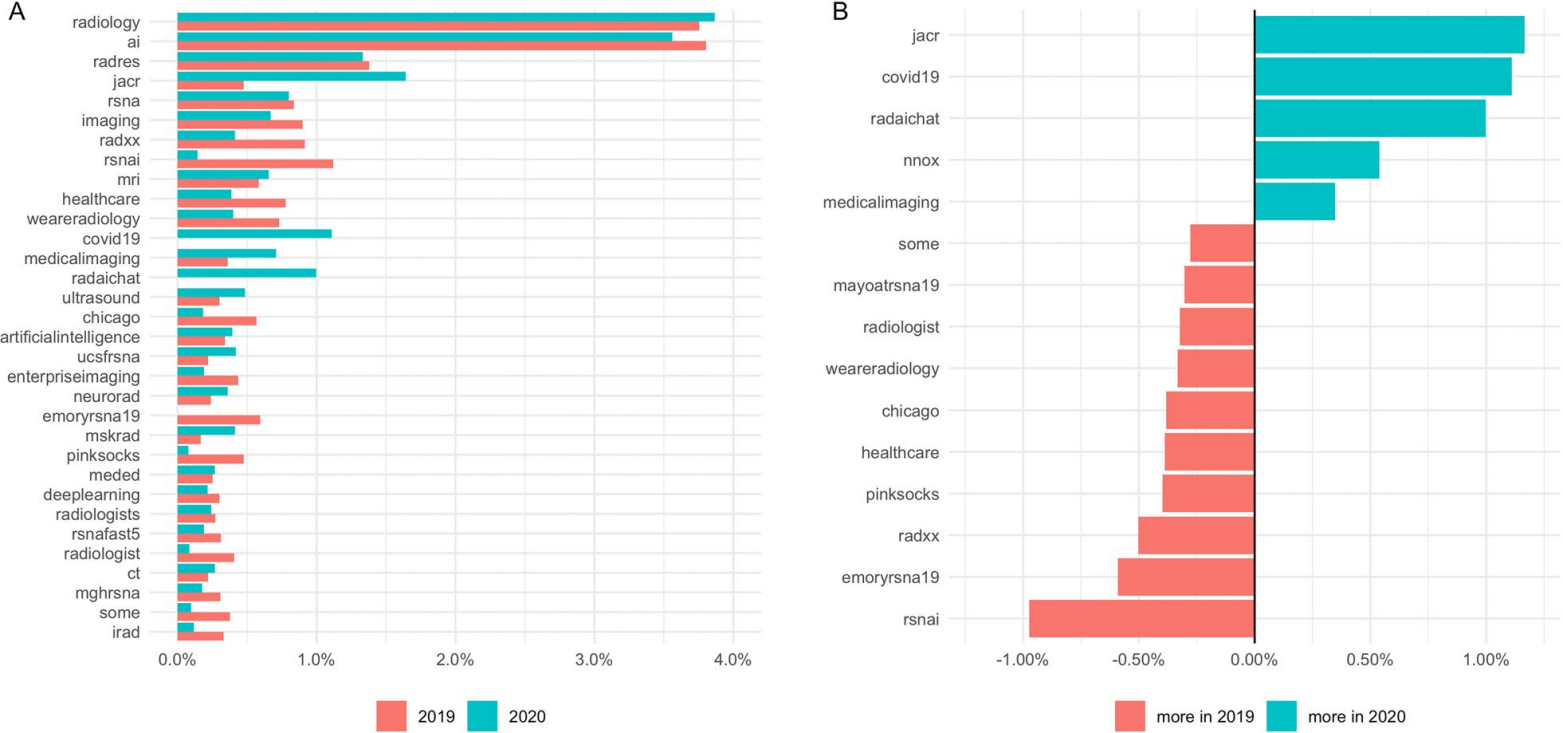
Hashtag frequencies. **A** Frequency of the most-used hashtags in 2019 and 2020 (excluding the congress hashtags #rsna19, #rsna2019, #rsna20, #rsna2020). The percentage share of individual hashtags in the total volume of hashtags during a year is shown. **B** Illustrates the largest absolute differences of hashtag frequencies in 2019 compared to 2020 in relation to total tweet count (total number of #radxx 2019: 254, 2020: 63; #weareradiology 2019: 203, 2020: 61; #pinksocks 2019: 132; 2020: 12; #some 2019: 104, 2020: 15; #chicago 2019: 157, 2020: 28; #jacr 2019: 132, 2020: 250; #nnox 2019: 0, 2020: 82; #radaichat 2019: 0, 2020: 152; #covid-19 2019: 0, 2020: 169)

To examine hashtag usage, we determined the most frequently used hashtags in relation to the total number of tweets in each year (Fig. 1A) and visualized major changes in hashtag frequency across years (Fig. 1B). Clearly, artificial intelligence (AI) was the leading topic in both years, especially when considering that many other hashtags were also related to AI. Notably, hashtags for social initiatives such as “#radxx,” and “#weareradiology” (social initiatives for gender equality and diversity in radiology), “#pinksocks” (a social initiative), “#some” (abbreviation for social media) or “#chicago” (location of the physical RSNA conference) were used more frequently in 2019 compared to 2020. In contrast, a Twitter-based chat for radiologists on Twitter called “radaichat” conducted by the journal “Radiology—Artificial Intelligence” was more frequently used in 2020. COVID-19-related hashtags were obviously only used in 2020. In addition, “#jacr” (Journal of the American College of Radiology) and “#nnox” (popular stock of Nano-X Imaging Ltd that went public in 2020) were also used more often in 2020 (Fig. 1B).

### Hashtag pairs

**Fig. 2:**
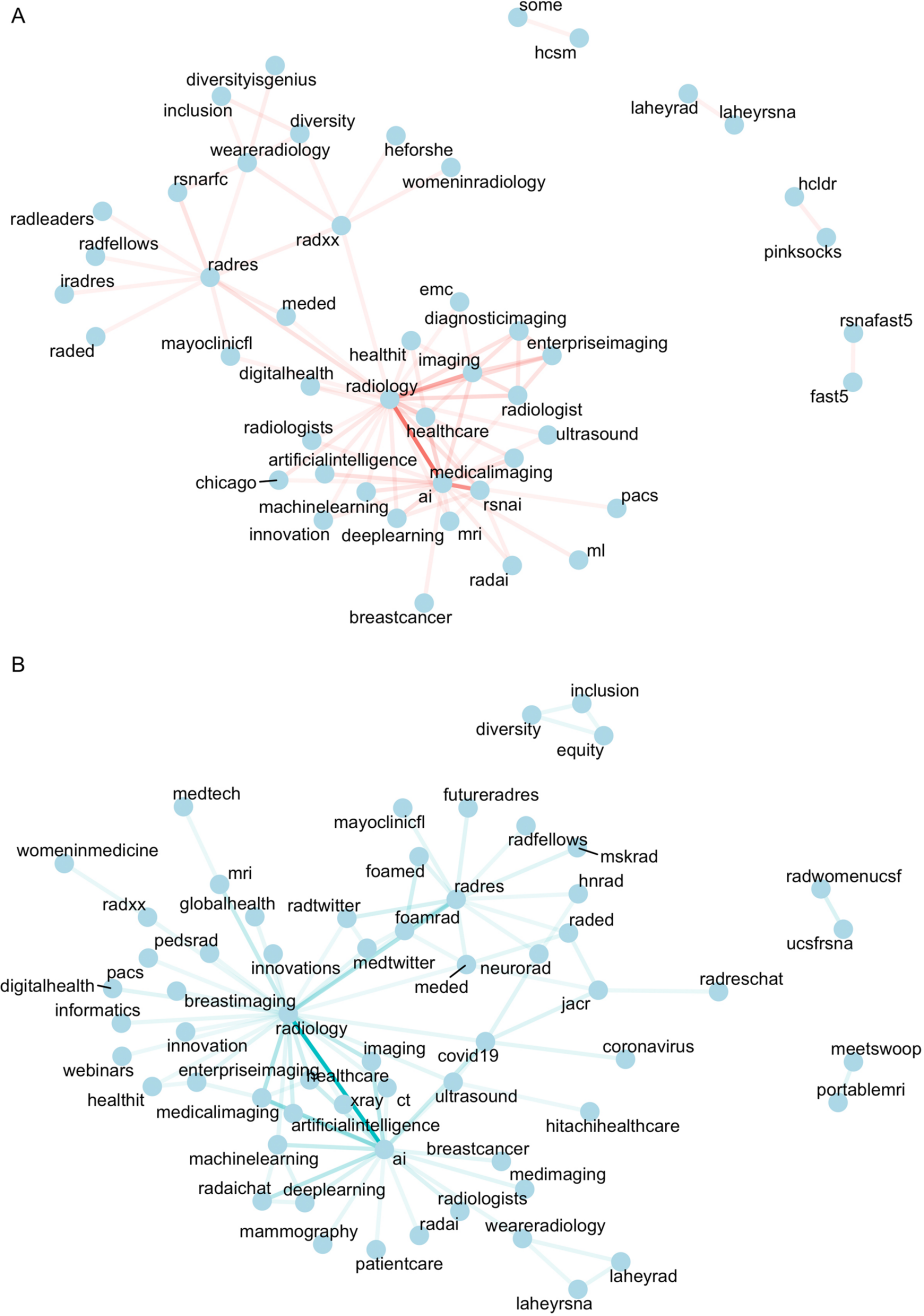
Hashtag co-occurrence. Network graph of most frequently co-occurring hashtags in 2019 (**A**) and 2020 (**B**) (excluding the congress hashtags #rsna19, #rsna2019, #rsna20, #rsna2020). Strength of connection indicates frequency of co-occurrence

In addition, we analyzed which pairs of hashtags most frequently co-occurred across both years (Fig. 2). In both years, the strongest connection was between the hashtags “#ai” and “#radiology.” Mirroring our result in Fig. 1, there were more co-occurring hashtags related to social initiatives (e.g., gender equality and diversity (“#radxx,” “#womeninradiology,” “#inclusion,” “#diversityisgenius,” “#weareradiology,” “#heforshe”)) in 2019 than in 2020. Additionally, we noticed a high co-occurrence of Spanish language hashtags in 2020; however, this appeared to be an artifact introduced by one highly active Spanish language user (“@droswaldoramos”). Consequently, we removed the tweets of this user from this part of our analysis.

## Discussion

Twitter has become one of the most important social media platforms for science and is heavily used during medical conferences. Therefore, the main goal of this study was to clarify whether Twitter activity and conference attendance are related.

Indeed, we found that there was a 43% decrease in attendance—from 51.800 in 2019 [19] to 29,339 attendees in 2020 [20]—matching a 46% decrease in total tweets and a 39% decrease in individual Twitter users between 2019 and 2020, indicating that Twitter activity may be a good surrogate parameter for attendance during these conferences. However, it is important to mention that further analysis of more conferences and related tweets is needed to underline and secure this finding. Interestingly, a previous study examined the Twitter activity during RSNA conferences, showing an 30% increase in Twitter activity between 2011 and 2012 [10], while there was a 9% attendance drop from 2011 to 2012. One reason for the results of Hawkins et al., which contradicts our results, could be the novelty of Twitter in science combined with skyrocketing growth rates of science tweets 10 years ago. Another reason for a shift of users from Twitter to other social media might be the political controversy regarding the presidential tweets of Donald Trump. Besides Twitter, LinkedIn is the most popular social media platform for professional content among Radiologists [18]. Therefore, it may be of interest in the future to compare radiologists' use of Twitter and LinkedIn.

Moreover, we were interested in the influence of a virtual conference on tweeting behavior and the structure of Twitter users. Many studies reported that virtual conferences attract higher number of attendees compared to in-person conferences [21–23]. Sarabipour et al. [22] examined multiple virtual medical conferences and argued that they are “more inclusive, more affordable, less time-consuming and more accessible worldwide.” In contrast, we found that the attendance and the tweeting activity (e.g., total tweets and retweets per tweet) during the virtual RSNA conference decreased compared to the in-person conference. In addition, topics related to social initiatives and gender equality were less discussed on virtual compared to the in-person RSNA conference. An important example is the decreased presence of the hashtag “radxx” during the virtual RSNA conference. The “#radxx” hashtag stands for a movement to empower women in radiology, informatics and IT management [24]. It was founded by a RSNA member (Dr. Geraldine McGinty) during the RSNA conference 2016. With 200 members in the community the “#radxx” movement shows the importance of gender equality for the RSNA. We found other hashtags that showed the same development and also stand for social and personal topics (e.g., “#weareradiology” and “#pinksocks”). We postulate that the decrease in social and personal topics during the virtual RSNA conference is caused by a lack of social interaction and interpersonal communication. In addition, this lack of social interaction and interpersonal communication reduced the opportunities to network, expressed by a smaller number of newly acquired followers among the Top 50 users during the virtual RSNA conference. Interestingly, professional topics were less strongly influenced by the virtual nature of the conference, as can be seen, among other things, from the great importance of AI in both years.

### Limitations

It is important to mention that this study is observational and cannot show causality between the concept of a virtual conference and lower attendance. There may be other possible reasons for the decrease in Twitter activity and attendance of the RSNA conference in 2020. Among those could be COVID-19-related changes of working environment and private lifestyle of people all over the world. Furthermore, this study only analyzed the hashtags of the tweets which are not always representative for the whole content. And importantly, more conferences need to be examined to clarify whether Twitter activity can serve as a surrogate parameter for conference attendance. Nonetheless, we believe that our study provides evidence for a relationship between Twitter activity and conference attendance and suggests decreased opportunities for social interaction at virtual conferences.

## Conclusions

All in all, this study provides evidence for decreased Twitter activity and different discussed topics during a virtual compared to an in-person medical conference. On top of that, this study presents that Twitter and conference attendance during medical conferences are highly related. Future studies may examine more medical conferences to prove that Twitter can serve as a surrogate parameter for conference attendance. Nevertheless, the integration of Twitter and a higher focus on interpersonal topics on medical conferences will be important for future (virtual) conferences.

## Data Availability

The datasets used and/or analyzed during the current study are available from the corresponding author on reasonable request.

## Abbreviations

AI: Artificial intelligence
ANOVA: Analysis of variance
COVID-19: Coronavirus Disease 2019
RSNA: The Radiological Society of North America

## References

[1] Raghupathi W, Raghupathi V (2014). Big data analytics in healthcare: promise and potential. Health Inf Sci Syst.

[2] Bundy JJ, Hage AN, Chick JFB, Srinivasa RN, Patel N, Johnson E (2018). Radiology: a 7-year analysis of radiology-associated hashtags. Curr Probl Diagn Radiol.

[3] Rostampour S, Hamady MS, Alsafi A (2020). To tweet or not to tweet? A look at radiology societies’ use of Twitter. Cardiovasc Intervent Radiol.

[4] Nason GJ, O’Kelly F, Kelly ME, Phelan N, Manecksha RP, Lawrentschuk N (2015). The emerging use of Twitter by urological journals. BJU Int.

[5] Stellrecht E, Hendrix D (2016). AAHSL Twitter use from 2007 to 2014: an exploratory analysis. Med Ref Serv Q.

[6] Pershad Y, Hangge P, Albadawi H, Oklu R (2018). Social medicine: Twitter in healthcare. J Clin Med.

[7] Miles RC, Patel AK (2019). The radiology Twitterverse: a starter’s guide to utilization and success. J Am Coll Radiol.

[8] Stoneman S, Hiremath S (2020). Twitter-based journal clubs: bringing critical appraisal to the social table. Semin Nephrol.

[9] Kalia V, Ortiz DA, Patel AK, Moriarity AK, Canon CL, Duszak R (2018). Leveraging Twitter to maximize the radiology meeting experience. J Am Coll Radiol.

[10] Hawkins CM, Duszak R, Rawson JV (2014). Social media in radiology: early trends in Twitter microblogging at radiology’s largest international meeting. J Am Coll Radiol.

[11] Cevik M, Ong DSY, Mackenzie G (2019). How scientists and physicians use Twitter during a medical congress. Clin Microbiol Infect.

[12] Attai DJ, Radford DM, Cowher MS (2016). Tweeting the meeting: twitter use at the American Society of Breast Surgeons annual meeting 2013–2016. Ann Surg Oncol.

[13] McKendrick DRA, Cumming GP, Lee AJ (2012). Increased use of Twitter at a medical conference: a report and a review of the educational opportunities. J Med Internet Res.

[14] Neill A, Cronin JJ, Brannigan D, O’Sullivan R, Cadogan M (2014). The impact of social media on a major international emergency medicine conference. Emerg Med J.

[15] Pemmaraju N, Mesa RA, Majhail NS, Thompson MA (2017). The use and impact of Twitter at medical conferences: best practices and Twitter etiquette. Semin Hematol.

[16] Djuricich AM, Zee-Cheng JE (2015). Live tweeting in medicine: “Tweeting the meeting”. Int Rev Psychiatry.

[17] Rosenkrantz AB, Hawkins CM (2017). Use of Twitter polls to determine public opinion regarding content presented at a major national specialty society meeting. J Am Coll Radiol.

[18] Ranschaert ER, Van Ooijen PM, McGinty GB, Parizel PM (2016). Radiologists' usage of social media: results of the RANSOM survey. J Digit Imaging.

[19] Radiological Society of North America. https://www.rsna.org/. Accessed 28 Feb 2021

[20] RSNA says 29K attended 2020 virtual meeting. Aunt Minnie. https://www.auntminnie.com/index.aspx?sec=ser&sub=def&pag=dis&ItemID=131127. Accessed 8 Mar 2021

[21] Castelvecchi D (2020). “Loving the minimal FOMO”: first major physics conference to go virtual sees record attendance. Nature.

[22] Sarabipour S (2020). Virtual conferences raise standards for accessibility and interactions. Elife.

[23] Gone virtual: lessons from ICLR2020 | by ICLR | Medium. https://iclr-conf.medium.com/gone-virtual-lessons-from-iclr2020-1743ce6164a3. Accessed 4 Mar 2021

[24] Retrouvey M, Keefe B, Kotsenas A, McGinty G, Patel AK (2018). Women in radiology: creating a global mentorship network through social media. J Am Coll Radiol.

